# PHGDH supports liver ceramide synthesis and sustains lipid homeostasis

**DOI:** 10.1186/s40170-020-00212-x

**Published:** 2020-06-15

**Authors:** Yun Pyo Kang, Aimee Falzone, Min Liu, Paloma González-Sánchez, Bo-Hyun Choi, Jonathan L. Coloff, James J. Saller, Florian A. Karreth, Gina M. DeNicola

**Affiliations:** 1 Department of Cancer Physiology, H. Lee Moffitt Cancer Center and Research Institute, Tampa, FL USA; 2 Proteomics and Metabolomics Core Facility, H. Lee Moffitt Cancer Center and Research Institute, Tampa, FL USA; 3 Department of Physiology and Biophysics, University of Illinois Cancer Center, University of Illinois at Chicago, Chicago, IL USA; 4 Department of Anatomic Pathology, H. Lee Moffitt Cancer Center and Research Institute, Tampa, FL USA; 5 Department of Molecular Oncology, H. Lee Moffitt Cancer Center and Research Institute, Tampa, FL USA

**Keywords:** PHGDH, Serine, Ceramide, Triacylglycerol, Mouse model

## Abstract

**Background:**

d-3-phosphoglycerate dehydrogenase (PHGDH), which encodes the first enzyme in serine biosynthesis, is overexpressed in human cancers and has been proposed as a drug target. However, whether PHGDH is critical for the proliferation or homeostasis of tissues following the postnatal period is unknown.

**Methods:**

To study PHGDH inhibition in adult animals, we developed a knock-in mouse model harboring a PHGDH shRNA under the control of a doxycycline-inducible promoter. With this model, PHGDH depletion can be globally induced in adult animals, while sparing the brain due to poor doxycycline delivery.

**Results:**

We found that PHGDH depletion is well tolerated, and no overt phenotypes were observed in multiple highly proliferative cell compartments. Further, despite detectable knockdown and impaired serine synthesis, liver and pancreatic functions were normal. Interestingly, diminished PHGDH expression reduced liver serine and ceramide levels without increasing the levels of deoxysphingolipids. Further, liver triacylglycerol profiles were altered, with an accumulation of longer chain, polyunsaturated tails upon PHGDH knockdown.

**Conclusions:**

These results suggest that dietary serine is adequate to support the function of healthy, adult murine tissues, but PHGDH-derived serine supports liver ceramide synthesis and sustains general lipid homeostasis.

## Background

The amino acid l-serine is derived from the diet, protein degradation, hydroxymethylation of l-glycine, and/or de novo biosynthesis. In the first step of de novo l-serine biosynthesis, d-3-phosphoglycerate dehydrogenase (PHGDH) catalyzes the formation of 3-phosphohydroxypyruvate from the glycolytic intermediate 3-phosphoglycerate. PHGDH activity is increased in many cancer cells as a consequence of genomic amplification, transcriptional upregulation, posttranslational modification, and allosteric regulation [7, 9, 15, 22, 24, 32, 34, 40]. Cancer cells with increased PHGDH activity are more dependent on PHGDH for proliferation, suggesting PHGDH is an attractive target for cancer therapy. Indeed, multiple PHGDH inhibitors have been developed, although none have reached the clinic to date.

While the contribution of PHGDH and serine to cancer cell metabolism has been well studied [8, 20, 25–27, 33, 39, 46, 47], less is known about the importance of PHGDH in normal tissues. Whole animal PHGDH deletion is embryonic lethal in mice as a consequence of overall developmental retardation and brain defects [48]. Brain-specific deletion dramatically reduces l-serine and d-serine levels in the cerebral cortex and hippocampus [45], leads to the development of postnatal microcephaly [45], and results in the accumulation of toxic deoxysphingolipids in the hippocampus [11]. Targeted deletion of PHGDH in endothelial cells is similarly lethal shortly after birth as a consequence of vascular defects due to compromised heme synthesis and mitochondrial function [42]. By contrast, PHGDH deletion in adipocytes presents with no overt phenotype, but improves glucose tolerance upon diet-induced obesity [31]. However, whether PHGDH is critical for the proliferation or homeostasis of other tissues following the postnatal period is unknown.

To study how PHGDH inhibition affects the functions of normal tissues in adult animals, we developed a knock-in mouse model harboring a PHGDH shRNA under the control of a doxycycline-inducible promoter. With this model, PHGDH depletion can be globally induced in adult animals, while sparing the brain due to poor doxycycline penetration. We find that PHGDH depletion is well tolerated in multiple highly proliferative cell compartments, with no overt phenotypes observed following knockdown. Further, PHGDH knockdown leads to a reduction in ceramide levels and an increase in triglyceride long chain (LC) polyunsaturated fatty acid (PUFA) content. These results suggest that dietary serine is adequate to support the function of healthy, adult murine tissues.

## Methods

### Reagents

HPLC grade chloroform (Sigma-Aldrich, 650498-1 L) and methanol (Sigma-Aldrich, 34860-1 L-R) were obtained from Sigma-Aldrich. HPLC grade water (W5-1) was from Fisher Scientific. The following internal standards were used in targeted lipidomics analyses: Cer/Sph mixture II (contains Cer(d18:1/12:0), cat #LM6005, Avanti Polar Lipids)**,**d_7_-sphinganine (860658, Avanti Polar Lipids), d_3_-deoxysphinganine (860474, Avanti Polar Lipids)**,** C12-doxCer [Cer(m18:1/12:0)] (860455P-1 mg, Avanti Polar Lipids)**,** and C12-dihydro-doxCer [Cer(m18:0/12:0)] (860481P-1 mg, Avanti Polar Lipids). The following internal standards were used in metabolomics analyses: d_3_-serine (DLM-582-0.1, Cambridge Isotope Laboratories) and the Metabolomics Amino Acid Mix Standard (contains ^13^C_3_, ^15^n-serine, cat #MSK-A2-1.2, Cambridge Isotope Laboratories).

### shRNA validation

NIH3T3s (ATCC) and Lenti-X 293 T cells (Clontech) were grown in DMEM supplemented with 10% FBS. shRNAs were cloned into the LT3GEPIR vector [12] for shRNA testing. Lentivirus was produced in 293 T cells using helper plasmids pCMV-dR8.2 dvpr (addgene #8455) and pCMV-VSV-G (addgene #8454). NIH3T3 cells were infected with lentivirus encoding doxycycline-inducible shRNA constructs at an MOI of 0.2 for single-copy integration. Cells were treated with 1 μg/mL doxycycline for 6 days, and PHGDH expression was determined by western blot. shRNA #3 (targeting 5′-ACCTGAACTAATACCTAGTAA-3′) was selected and cloned into the col1A1 targeting vector cTGME for ESC targeting.

### Mice

To generate shPHGDH mice, C10 murine ES cells [3] were targeted by recombination-mediated cassette exchange as previously described [35] and selected with hygromycin. Positive clones were screened by PCR and injected into blastocysts. Mice expressing a Renilla luciferase control shRNA (shREN) [35], and ROSA26-CAGs-rtTA3 mice [10] were obtained from Dr. Lukas Dow (Weill Cornell Medicine). Mice were housed and bred in accordance with the ethical regulations and approval of the IACUC (protocol #IS00003893R). Mice were maintained on a mixed C57B6/129 background. shPHGDH, R26-CAGs-rtTA3, and shREN, R26-CAGs-rtTA3 mice were given a doxycycline-containing diet (200 ppm, Envigo), which was replaced weekly.

### Blood counts

Blood was collected from submandibular vein into Eppendorf tubes containing EDTA. Samples were completely mixed and stored at room temperature until analysis. All blood samples were analyzed within 4 h of collection. Complete blood counts were analyzed with the Procyte Dx Hematology Analyzer (IDEXX).

### Oral glucose tolerance test

Mice were placed on doxycycline diet for approximately 100 days before use. Mice were fasted overnight, followed by oral administration of 2 g/kg d-glucose solution. Blood was serially sampled at the indicated time points, and glucose levels were determined with the OneTouch Ultra Mini Blood Glucose Monitoring System.

### Immunohistochemistry

Tissues were fixed in 10% formalin overnight before embedding in paraffin and sectioning by IDEXX BioAnalytics. Sections were de-paraffinized in xylene, followed by rehydration in a graded alcohol series. Heat-mediated antigen retrieval (microwave, 12.5 min on high) was performed in 10 mM citrate buffer (pH 6.0). Endogenous peroxidase activity was quenched with 3% hydrogen peroxide in tap water for 5 min. Immunohistochemical staining was performed with the ImmPRESS HRP anti-rabbit kit according to manufacturer’s instructions (Vector Labs), followed by incubation with DAB substrate (Vector Labs). The following antibodies were used: Ki-67 (1:400; Cell Signaling, 12202).

### LC-MS based Lipidomics

The liver or brain tissue samples were homogenized with a pre-chilled BioPulverizer (59012MS, BioSpec) and then placed on dry ice. The chloroform:methanol extraction solvent (v:v = 1:2) containing 5 nM Cer (m18:1/12:0), 5 nM Cer (m18:0/12:0), 12.5 nM d_3_-deoxysphinganine, and 50 nM Cer (d18:0/12:0) internal standards was added to homogenates to meet 50 mg/mL. The samples were then sonicated in ice cold water using Bioruptor^TM^ UCD-200 sonicator for 5 min (30 s sonication and 30 s rest cycle; high voltage mode). For serum samples, 75 μL of chloroform:methanol extraction solvent (v:v = 1:2) containing 5 nM Cer (m18:1/12:0), 50 nM of Cer (d18:0/12:0), and 12.5 nM d_3_-deoxysphinganine internal standards was added to 20 μL of mouse serum. After shaking (1400 rpm, 20 °C, 5 min), the extracts were cleared by centrifugation (17,000 g, 20 °C, 10 min), and the lipids in the supernatant were analyzed by LC-MS.

The HPLC condition was adapted from a previous study [17]. Chromatographic separation was conducted on a Brownlee SPP C18 column (2.1 mm × 75 mm, 2.7 μm particle size, Perkin Elmer, Waltham, MA) using mobile phase A (100% H2O containing 0.1% formic acid and 1% of 1 M NH4OAc) and B (1:1 acetonitrile:isopropanol containing 0.1% formic acid and 1% of 1 M NH4OAc). The gradient was programmed as follows: 0–2 min 35% B, 2–8 min from 35 to 80% B, 8–22 min from 80 to 99% B, 22–36 min 99% B, and 36.1–40 min from 99 to 35% B. The flow rate was 0.400 mL/min. The parallel reaction monitoring (PRM) approach was applied for quantification of deoxysphingolipids in positive ESI mode. The MS and MS/MS m/z values of deoxysphingolipids for PRM analysis were adapted from a previous study [11] (Supplementary Table 1). The collision energies of deoxysphingolipid species for PRM approach were set as following: 40 for doxCer and dihydro-doxCer and 15 for deoxysphinganine. For non-targeted lipidomics, the data-dependent MS^2^ scan conditions were applied: the scan range was from m/z 70–1000, resolution was 120,000 for MS, and 30,000 for DDMS^2^ (top 10), and AGC target was 3E6 for full MS and 1E5 for DDMS^2^, allowing ions to accumulate for up to 200 ms for MS and 50 ms for MS/MS. For MS/MS, the following settings are used: isolation window width 1.2 m/z with an offset of 0.5 m/z, stepped NCE at 10, 15, and 25 a.u., minimum AGC 5E2, and dynamic exclusion of previously sampled peaks for 8 s.

For targeted lipidomics, deoxysphingolipid and ceramide peaks identified by MS^2^ were manually integrated using the Thermo Xcaliber Qual Browser. The quantification was based on previous methods [11, 36]. For non-targeted lipidomics, lipid peaks including triacylglycerols and ceramides were identified, aligned, and exported using MS-DIAL [41]. The data were further normalized to the median value of total lipid signals. Only lipids fully identified by MS^2^ spectra were included in the analysis.

### Serum serine quantification by GC-MS

50 μL of serum was extracted at − 80 °C for 15 min with 450 μL of 88.8% MeOH containing 205 μM d_3_-serine. Following centrifugation (17,000 g, 4 °C, 20 min), 100 μL of supernatant was transferred to a new Eppendorf tube and then dried by centrifugation under vacuum (SpeedVac, Thermo Scientific). The dried pellets were further derivatized as previously described [6]. Briefly, the pellets were derivatized by 50 μL of methoxylamine hydrochloride (40 mg/mL in pyridine) at 30 °C for 90 min. The derivatized solution was then mixed with 70 μL of ethyl-acetate in a glass vial, and the mixture was further derivatized with 80 μL of MSTFA + 1%TMCS solution at 37 °C for 30 min. The final derivatized solution was then analyzed by GC-MS as previously described [18] using an MS scan range from 50 to 600 m/z. The derivatized serine (3TMS, 306 m/z) and d_3_-serine (3TMS, 309 m/z) peaks were extracted and integrated manually using the Agilent MassHunter Qualitative Analysis Software (Version B.07.00). The quantification was based on previous methods [4].

### Liver and brain serine quantification by LC-MS

After pulverization of liver and brain tissue with a pre-chilled BioPulverizer (59012MS, BioSpec), the extraction solvent (80% MeOH containing 2.49 μM ^13^C_3_, ^15^n-serine, − 80 °C) was added for a final concentration of 50 mg tissue/mL and incubated for 24 hr at − 80 °C. The metabolite extracts were centrifuged (17,000 g, 4 °C, 20 min), and the supernatant was analyzed by LC-MS as previously described [18]. Data was acquired in ESI-positive mode. For targeted quantification of serine, ^12^c-serine and ^13^C_3_, ^15^n-serine peak areas were manually integrated by EL-Maven (Version 0.6.1). Quantification was based on previous methods [4].

### In vivo glucose tracing

In vivo U-^13^C-glucose infusions were performed on shPHGDH and shREN mice implanted with a catheter in the jugular vein. Catheterized mice were fasted overnight by transferring to new cages without food and infused the following morning starting around 8 AM. Infusions were performed on awake mice on a tether/swivel system (Instech Laboratories) to allow free movement during the procedure and prevent anesthesia-induced changes in metabolism. A solution of 6 mg/g body weight/mL U-^13^C-glucose was prepared in sterile saline. Mice were infused at a rate of 100ul/min for the first minute and then 3ul/min for the next 2.5 h. At the end of the infusion, blood was collected via the submandibular vein into serum separator tubes (BD, cat# 365967) and placed on ice until centrifugation for serum collection. Next, mice were euthanized by the cervical dislocation, liver, pancreas, and brain quickly dissected, and tissues were snap frozen in liquid nitrogen. Serum and tissue samples were kept at − 80 °C until analysis.

Tissues were pulverized with a pre-chilled BioPulverizer (59012MS, BioSpec), and the extraction solvent (80% MeOH, − 80 °C) was added for a final concentration of 50 mg tissue/mL, followed by incubation for 24 hr at − 80 °C. For serum metabolite extraction, 90 μL of extraction solvent (88.8% MeOH, − 80 °C) was added to 10 μL of serum, vortexed for 10 sec, and incubated for 15 min at − 80 °C. The metabolite extracts were centrifuged (17,000 g, 4 °C, 20 min), and the supernatants were analyzed by LC-MS as previously described [18]. Data was acquired in ESI-negative mode. For liver tissue, which had a small amount of *M* + 3 serine, targeted analysis of ^13^c-labeled serine was performed. The ions for selective ion monitoring (SIM) approach were selected at positive mode as following: 106 [*M* + 0 + H]^+^, 107 [*M* + 1 + H]^+^, 108 [*M* + 2 + H]^+^, and 109 [*M* + 3 + H]^+^_._ The labeled or unlabeled peak areas were integrated using EL-Maven (Version 0.6.1) or Thermo Xcaliber Qual Browser. Data were corrected for natural occurring isotope abundance using the IsoCor Software [28].

### Immunoblotting

Tissue lysates were prepared by dounce homogenization in RIPA buffer (20 mM Tris-HCl [pH 7.5], 150 mM NaCl, 1 mM EDTA, 1 mM EGTA, 1% NP-40, 1% sodium deoxycholate) containing protease inhibitors (Roche complete). Protein concentrations were determined by the DC protein assay (Bio-Rad). Lysates were mixed with 6× sample buffer containing β-ME and separated by SDS-PAGE using NuPAGE 4–12% Bis-Tris gels (Invitrogen), followed by transfer to 0.45 μm nitrocellulose membranes (GE Healthcare). The membranes were blocked in 5% non-fat milk in TBST, followed by immunoblotting with the following antibodies: PHGDH (Sigma-Aldrich, HPA021241-100), PHGDH (Cell Signaling, 13428)–liver only, GFP (Cell Signaling, 2956), HSP90 (Cell Signaling, 4874), and β-actin (Thermo Fisher, AM4302, clone AC-15).

### Statistical analysis

Data were analyzed using a two-sided unpaired Student’s *t* test or Kaplan-Meier analysis as noted. GraphPad Prism 7 and 8 software were used for all statistical analyses, and values of *p* < 0.05 were considered statistically significant (*p < 0.05; **p < 0.01; ***p < 0.001).

## Results

### Generation of an inducible model for systemic PHGDH knockdown

**Fig. 1:**
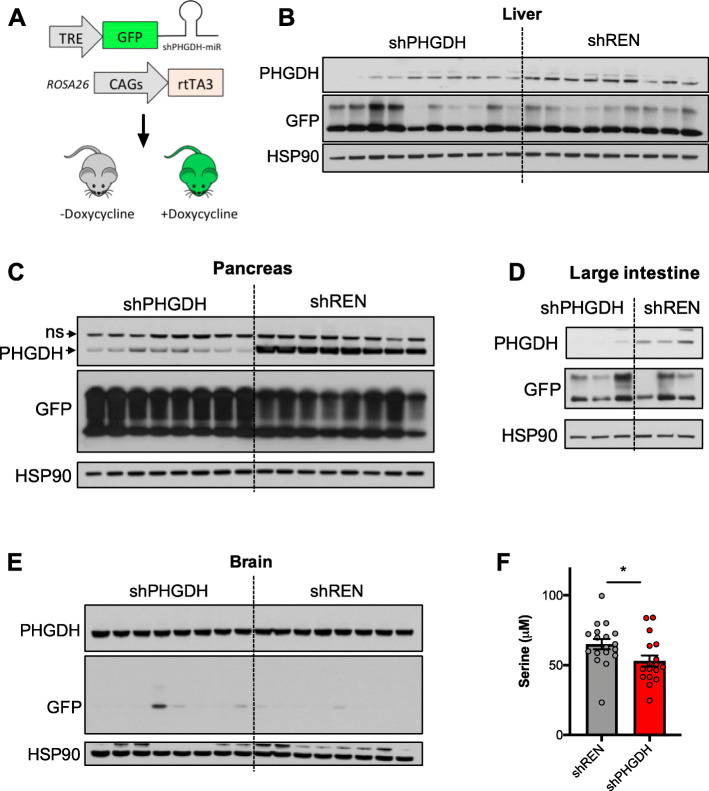
Generation of an inducible model for systemic PHGDH knockdown. **a** Schematic representation of the inducible shRNA system. **b**–**e** Western blot analysis of PHGDH, GFP, and HSP90 protein levels in the liver (**b**), pancreas (**c**), large intestine (**d**), and brain (**e**) of shPHGDH and shREN mice. ns, non-specific band. Mice were placed on a 200-ppm doxycycline diet for 4–8 months. **f** Serum serine concentrations of 8-month-old shREN (*N* = 16) and shPHGDH (*N* = 16) mice

In order to evaluate the requirement for serine biosynthesis in normal, proliferating adult tissues, we generated a mouse model in which an shRNA targeting PHGDH is linked to GFP and expressed in a doxycycline-inducible manner (Fig. 1a). To generate this model, we first tested the efficacy of single-copy shRNAs targeting mouse PHGDH in NIH3T3 cells (Supplementary Figure 1). shRNA #3 was selected, cloned into a col1A1 targeting vector for recombination-mediated cassette exchange in embryonic stem cells, and used to generate shPHGDH mice. Mice expressing Renilla luciferase targeting shRNA (shREN) were used as controls [35].

shREN and shPHGDH mice were crossed with a ubiquitous reverse tetracycline transactivator allele (rtTA3) to allow for whole body expression of the shRNA upon doxycycline administration. We selected the CAGs-rtTA3 allele, which demonstrated robust rtTA3 expression and activity in the pancreas, liver, kidney, small intestine, large intestine, skin, thymus, and bone marrow, although limited activity was observed in the spleen [10]. Further, because doxycycline levels achieved in the mouse brain are an order of magnitude lower than in plasma [23], this model is expected to spare the brain and avoid the previously reported brain toxicity associated with PHGDH knockout. shRNA, rtTA3 dual allele mice were placed on a 200 ppm doxycycline-containing diet containing serine and glycine. As expected, western blot analysis of PHGDH expression in tissues revealed that PHGDH knockdown was achieved in the pancreas, liver, and large intestine (Fig. 1b–d), but was absent in the brain and spleen (Fig. 1e, Supplemental Figure 2). Serum serine levels were decreased by approximately 20% following PHGDH knockdown (Fig. 1f), with the remaining 80% likely accounted for by dietary serine and glycine. These results demonstrate that our inducible PHGDH knockdown model spares brain and spleen PHGDH but diminishes PHGDH in other tissues where rtTA3 is robustly expressed, resulting in a reduction in circulating serine.

### PHGDH knockdown impairs serine synthesis in vivo

**Fig. 2:**
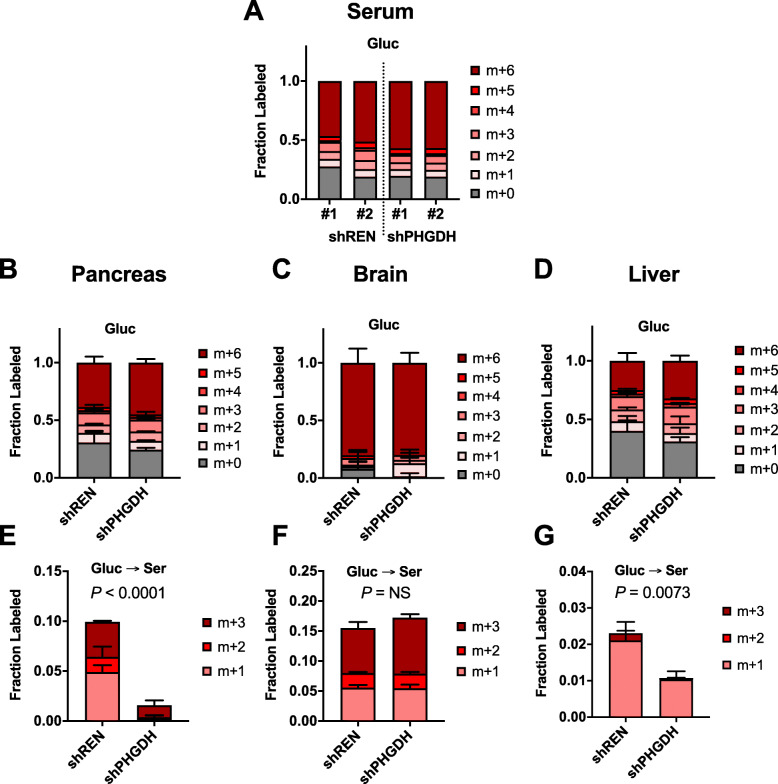
PHGDH knockdown impairs serine synthesis in vivo. **a** Fraction serum glucose labeling of shREN and shPHGDH mice infused with U-^13^C-glucose for 2.5 h. [^13^C]-label is denoted by the increase in mass (*M*) from *M* + 0 to *M* + *n,* where *n* denotes the number of labelled carbons. In addition to the expected 6 labeled carbons (*M* + 6), other isotopologues indicative of gluconeogesis were observed. **b**–**d** Fraction glucose labeling of shREN and shPHGDH mouse pancreas (**b**), brain (**c**), and liver (**d**). Data represent the average and SD (*n* = 4 tissue samples from the 2 mice from [A] for each group). **e**–**g** Fraction serine labeling of shREN and shPHGDH mouse pancreas (**e**), brain (**f**), and liver (**g**). Data represent the average and SD (*n* = 4 tissue samples from the 2 mice from [A] for each group). Total fraction labeling was compared with the Student *t* test. NS not significant. In addition to the expected 3 labeled carbons (*M* + 3), *M* + 1 labeling occurs as a consequence of folate cycle activity

We next validated that PHGDH knockdown impaired serine synthesis in vivo. To this end, we performed in vivo glucose tracing using uniformly labeled ^13^C-glucose (U-^13^C-glucose), which is metabolized to ^13^C-3-phosphoglycerate via glycolysis and subsequently to ^13^C-serine via PHGDH and the serine synthesis pathway. shPHGDH and shREN mice were implanted with jugular catheters to facilitate glucose infusions on active mice, allowed to recover, and fasted overnight prior to infusions. Infusion with ^13^C-glucose for 2.5 h resulted in robust labeling of serum glucose with no apparent difference in labeling between shPHGDH and shREN animals (Fig. 2a). This translated to robust tissue labeling of glucose in the pancreas, brain, and liver (Fig. 2b–d), with no difference in glucose labeling observed between shPHGDH and shREN animals. Liver glucose labeling was lower than other tissues, possibly due to gluconeogenesis in this organ. Importantly, shPHGDH animals demonstrated a reduction in serine labeling from glucose in the pancreas and liver, but not the brain (Fig. 2e–g). The reduction in serine labeling in the pancreas was greater than the liver, consistent with the robust knockdown achieved in the pancreas compared to liver (Fig. 1b, d). These results demonstrate that tissues with PHGDH knockdown have impaired serine synthesis proportional to the degree of protein knockdown achieved.

### PHGDH is not required for tissue proliferation and mouse viability

**Fig. 3:**
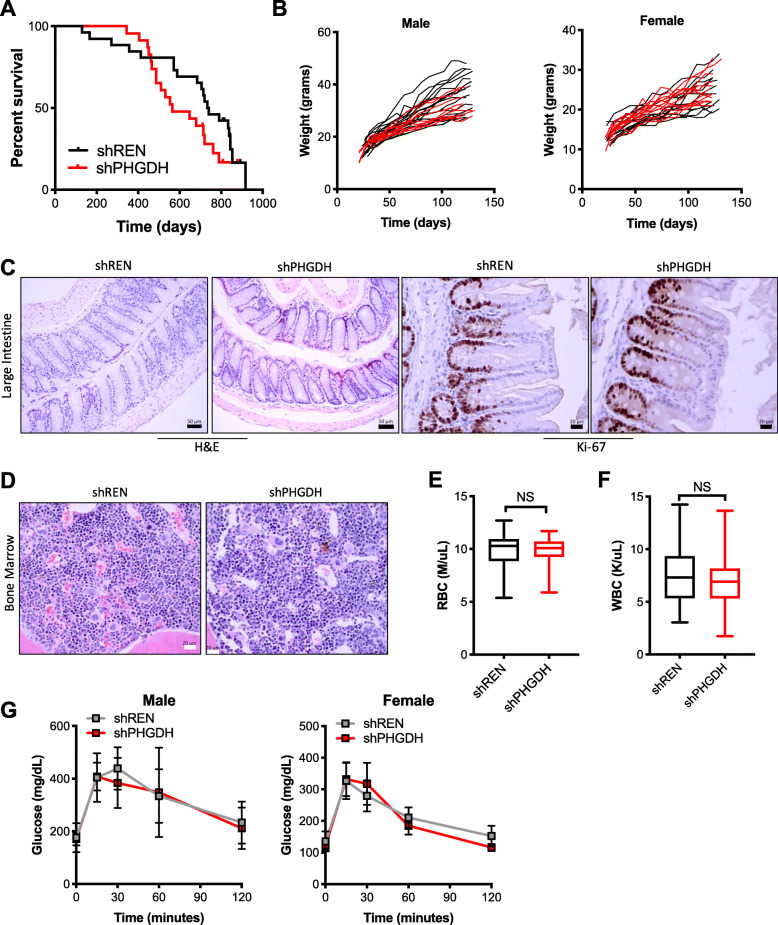
Systemic PHGDH depletion is non-toxic. **a** Overall survival of mice expressing shPHGDH (*N* = 23) or the control shRenilla (shREN, *N* = 26). **b** Weight of male and female mice expressing shPHGDH (male, *N* = 16; female, *N* = 15) or shREN (male, *N* = 13; female, *N* = 10). Mice were placed on doxycycline at weaning. **c** Representative hematoxylin and eosin stained (*N* = 10+) and Ki-67 immunostained (*N* = 5 each) large intestine sections from shPHGDH and shREN mice at endpoint. **d** Representative hematoxylin and eosin stained bone marrow sections from shPHGDH and shREN mice (*N* = 10+) at endpoint. **e** Red blood cell counts of shREN (*N* = 31) and shPHGDH (*N* = 33) mice at 8 months. **f** White blood cell counts of shREN (*N* = 31) and shPHGDH (*N* = 33) mice at 8 months. **g** Oral glucose tolerance test at 100 days on doxycycline. Male and female shPHGDH and shREN mice were challenged with 2 g/kg glucose at time = 0, and blood glucose levels were assayed at the indicated time points. Male shPHGDH (*N* = 7), male shREN (*N* = 7), female shPHGDH (*N* = 6), female shREN (*N* = 10)

We next assessed the consequence of PHGDH depletion on animal health and viability. Mice expressing shPHGDH were found to have a normal lifespan, with only a modest, non-significant reduction in survival compared to shREN mice (Fig. 3a). Because PHGDH has been associated with proliferation in neoplastic cells, we determined the effects of PHGDH silencing on the most proliferative tissues in adult mice. First, we examined the health of the intestine, which contains highly proliferative stem cells in the crypt, which must replace the entire epithelium every few days. We found that mice gained weight at normal rates, and intestines exhibited a normal morphology and proliferation rate (Fig. 3b, c). Next, we examined the hematopoietic cells in the bone marrow. We found no overtly abnormal phenotypes in the bone marrow of shPHGDH mice in the absence of stress (Fig. 3d). Further, shPHGDH mice had normal red blood cell and white blood cell counts (Fig. 3e, f). However, this may be explained by low basal PHGDH expression in the bulk bone marrow population, which was undetectable by western blot (not shown). Finally, because PHGDH knockdown was very robust in the pancreas, we examined the consequence of PHGDH depletion on glucose tolerance. shREN and shPHGDH mice were administered a bolus of glucose in an oral glucose tolerance test, and blood glucose was assayed over time. We found that glucose tolerance was not affected by PHGDH knockdown (Fig. 3g), suggesting that PHGDH is not required for normal pancreatic function. Further, PHGDH knockdown in the liver did not affect liver function as determined by blood markers for liver enzymes and other liver markers (Supplementary Figure 3). Collectively, these results demonstrate that PHGDH is not required for the cellular proliferation or normal function of multiple tissues in adult mice, which present with no overtly abnormal phenotypes upon PHGDH knockdown.

### shPHGDH mice do not exhibit deoxysphingolipid formation

**Fig. 4:**
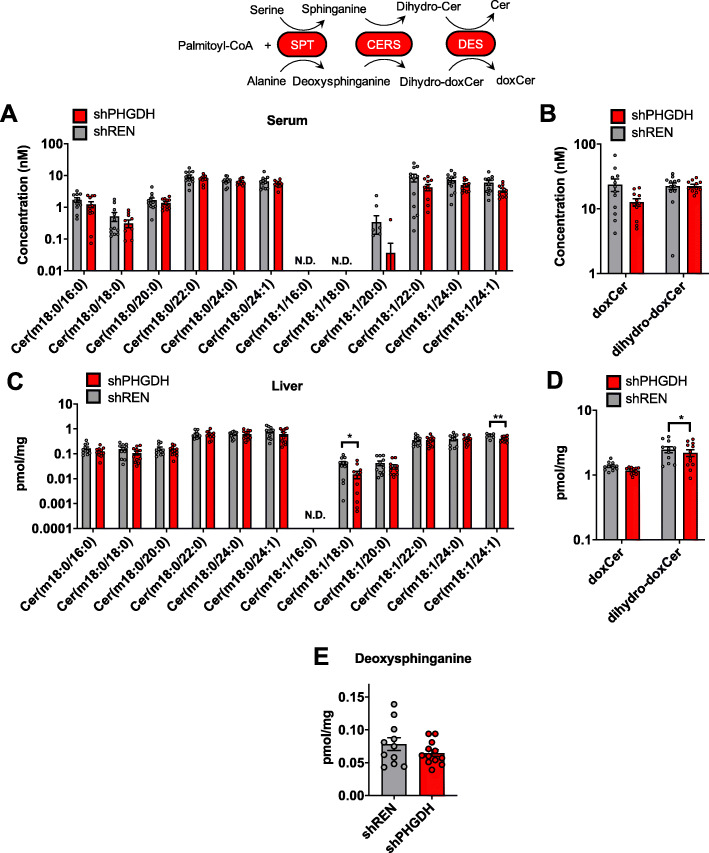
PHGDH knockdown does not promote deoxyshingolipid formation**. a** Concentration of individual deoxysphingolipids and dihydrodeoxysphingolipids in the serum of shREN and shPHGDH mice. **b** Total concentration of deoxysphingolipids (doxCer) and dihydrodeoxysphingolipids (dihydro-doxCer) in the serum of shREN and shPHGDH mice. **c** Quantity of deoxysphingolipids in the liver of shREN and shPHGDH mice. Quantities were normalized to mg of tissue. **d** Total quantity of deoxysphingolipids (doxCer) and dihydrodeoxysphingolipids (dihydro-doxCer) in the liver of shREN and shPHGDH mice. Quantities were normalized to mg of tissue. **e** Quantity of deoxysphinganine in the liver of shREN and shPHGDH mice. Quantities were normalized to mg of tissue. For **a**–**e**, 8-month-old shREN (*N* = 11) and shPHGDH (*N* = 12) mice were used for analysis

In addition to the importance of serine for the proliferation of neoplastic and non-neoplastic cells, low serine levels have been linked with toxic deoxysphingolipid formation in both serine deprivation experiments [13] and PHGDH knockout brains [11]. When serine levels are low, deoxysphingolipids are made via serine palmitoyltransferase (SPT) using alanine as a substrate instead of serine, thereby leading to deoxysphingolipid accumulation and cellular toxicity, particularly in the central nervous system. To examine the effect of PHGDH depletion on deoxysphingolipid metabolism, we first examined deoxysphingolipid levels in the circulation. However, we found no accumulation of individual (Fig. 4a) or total (Fig. 4b) dihydro-deoxy (dihydro-dox) or the deoxy (dox) sphingolipid species in the serum of shPHGDH mice. Similarly, liver deoxysphingolipid levels were not altered (Fig. 4c), with the levels of dihydro-deoxysphingolipids actually significantly lower upon PHGDH knockdown (Fig. 4d). Finally, the levels of the deoxysphingolipid precursor deoxysphinganine was not elevated in liver (Fig. 4e). These results demonstrate that the decrease in circulating serine following PHGDH knockdown is not sufficient to induce deoxysphingolipid formation.

### PHGDH knockdown decreases serum and liver ceramides

**Fig. 5:**
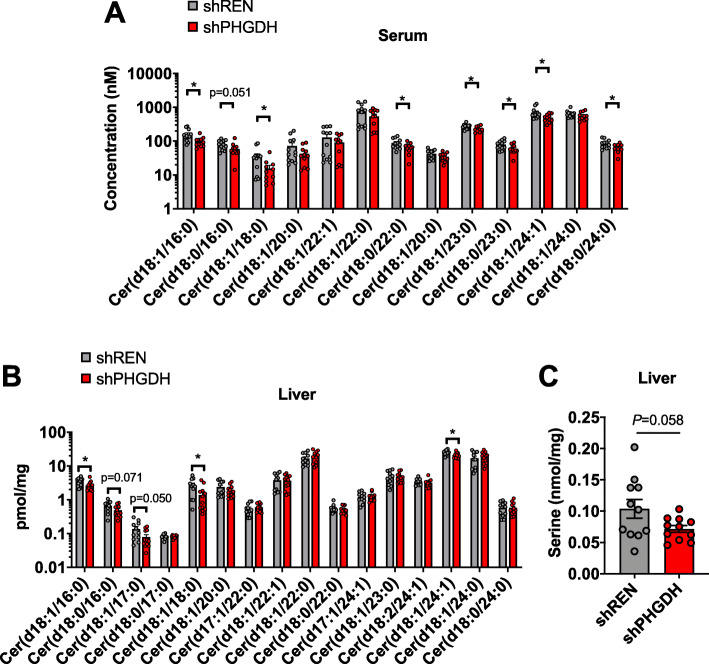
PHGDH knockdown depletes ceramides**. a** Concentration of individual ceramides in the serum of 8-month-old shREN (*N* = 12) and shPHGDH mice (*N* = 11). **b** Quantity of individual ceramides in the liver of 8-month-old shREN (*N* = 11) and shPHGDH (*N* = 12) mice. Quantities were normalized to mg of tissue. **c** Liver serine quantities of 8-month-old shREN (*N* = 11) and shPHGDH (*N* = 11) mice. Quantities were normalized to mg of tissue

Importantly, the canonical product of the SPT pathway, ceramide, can also be influenced by serine availability [14]. Interestingly, and in agreement with a previous report demonstrating that serine limitation depletes ceramide [14], we observe a decrease in many ceramide species in the serum of shPHGDH mice (Fig. 5a). Consistent with what was observed in the serum, liver ceramides were also significantly decreased (Fig. 5b). Analysis of liver serine levels revealed a decrease by approximately 20% (Fig. 5c), similar to what was observed in the serum (Fig. 1e). Collectively, these results demonstrate that PHGDH knockdown influences ceramide levels in vivo, consistent with prior studies describing effects of serine availability on ceramide metabolism.

### PHGDH knockdown alters triacylglycerol composition

**Fig. 6:**
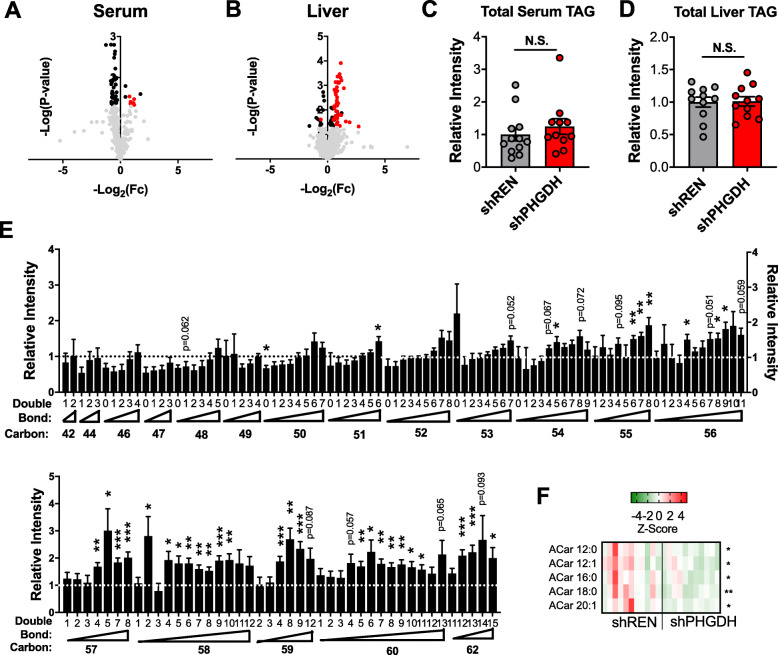
PHGDH knockdown alters liver lipid profiles. **a** Volcano plot of lipidomics analysis of shPHGDH (*N* = 11) serum compared to shREN (*N* = 12). Significant metabolites are in bold. Triacylglycerol species are indicated in red. **b** Volcano plot of lipidomics analysis of shPHGDH (*N* = 11) liver compared to shREN (*N* = 11). Significant metabolites are in bold. Triacylglycerol species are indicated in red. **c**, **d** Total triacylglycerol (TAG) levels in the serum (**c**) and liver (**d**) of shREN and shPHGDH mice. Levels are normalized to shREN. **e** Individual TAG species in the liver of shPHGDH mice compared to shREN. Levels are normalized to shREN. **f** Fatty acyl carnitine (Car) levels in the liver of shREN and shPHGDH mice from the analysis in (**b**). Only significant (*p* < 0.05) metabolites are shown

We next examined whether impaired sphingolipid synthesis influenced other lipid classes. Multiple lipid classes, such as sphingolipids, glycerophospholipids, and triacylglycerols, share similar fatty acid tails, with distinct lipid head groups. Lipidomics analysis of shREN and shPHGDH serum and liver revealed global lipid alterations (Fig. 6a, b, Supplementary Tables 2 and 3), suggesting a perturbation in lipid metabolism following PHGDH knockdown. In particular, we observed a significant accumulation of certain triacylglycerol (TAG) species, particularly in the liver (Fig. 6). However, total TAG levels in the serum and liver were unchanged (Fig. 6c, d). Rather, it was the composition of the TAG species that was altered. Specifically, accumulated TAG species were enriched in long chain (LC) polyunsaturated fatty acid (PUFA) tails, while short chain saturated TAGs were decreased (Fig. 6e). Further, lipidomics analysis of fatty acyl carnitine species revealed a significant decrease in C12:0-, C12:1-, C16:0-, C18:0-, and C20:1-carnitine species, suggesting a decrease in fatty acids availability for β-oxidation (Fig. 6f). In contrast, serine, ceramide, and TAG lipids were unaltered in the brain upon PHGDH knockdown (Supplemental Figure 4, Supplementary Table 4). These results demonstrate that PHGDH knockdown alters fatty acyl-carnitine levels and TAG composition in liver but does not affect total TAG levels.

## Discussion

Genetic deficiency in the de novo serine biosynthesis genes *PHGDH*, *PSAT1*, and *PSPH,* in humans causes Neu-Laxova syndrome (NLS), a very rare autosomal recessive congenital disorder [1, 37]. Severity is dictated by the degree of pathway activity loss, and most patients are affected from infancy. NLS patients present with central nervous system (CNS) symptoms including microcephaly, impaired motor function, epilepsy, and perinatal lethality. By contrast, we find that PHGDH depletion in non-cerebral tissues following the postnatal period results in no overt phenotype, which is consistent with the symptoms of PHGDH deficiency being predominantly localized to the CNS.

Our model is useful for the study of PHGDH in adult tissues, but it has some limitations. First, off-target effects are a concern with shRNAs. Second, knockdown is not complete in all tissues studied. While we could achieve better knockdown in some tissues with a diet containing 625 ppm doxycycline (not shown), concerns about the effects of doxycycline on metabolism led us to choose a lower concentration. Doxycyline suppresses the expression of oxidative phosphorylation genes and shifts metabolism to a more glycolytic phenotype [2], which could potentially mask mitochondrial metabolism-dependent phenotypes. Improved PHGDH depletion in some tissues may also be improved through the use of other rtTA3 alleles that have higher promoter activity in those tissues. Consequently, caution must be used when interpreting our results on the effect of PHGDH knockdown in all tissues because it is possible some cell types still have abundant PHGDH expression due to poor shRNA expression. Despite this limitation, our model is likely to more accurately model the effects of PHGDH inhibition in select tissues compared to whole body knockout due to the incomplete and transient nature of inhibition of enzymes by small molecules. While there have been many PHGDH inhibitors reported to date [29, 30, 33, 43, 44], only NTC-503, PKUMDL-WQ-2101, and PKUMDL-WQ-2201 have been used in vivo, and to our knowledge, their efficacy and long-term toxicity in various tissues have not been characterized. The future comparison between shRNA and pharmacological inhibition will provide important insight into the contribution of PHGDH to normal tissue homeostasis and metabolism.

Our findings suggest that PHGDH inhibitors that cannot cross the blood-brain barrier may be well tolerated, provided adequate serine and glycine are supplied through the diet. While liver lipid metabolism was altered, this did not appear to induce any pathology in the absence of stress. While we observed alterations in lipid levels in the serum and liver, we cannot exclude the possibility that decreased PHGDH-derived serine from another organ accounts for the decrease in serum and liver serine. It has been suggested that the kidney is the primary site of serine synthesis in humans and rats, with the liver contributing only under protein-deficient conditions [16]. Specialized diets lacking serine and glycine could be used to both compare with PHGDH knockdown, and reveal the importance of PHGDH to tissue homeostasis and metabolism under serine-limiting conditions. In addition, methionine/choline-deficient diets could be used, given the importance of serine to the folate and methionine cycles and high-fat diets could help dissect the lipid phenotype in the liver.

Our findings demonstrating that PHGDH knockdown results in a depletion of ceramides are very similar to findings seen with serine starvation in colon cancer cells, where ceramide depletion also led to loss of mitochondrial function, suggesting that mitochondrial function may also be impaired in our model as well. Interestingly, inhibition of mitochondrial function was recently found to induce the synthesis of highly unsaturated fatty acids (HUFA) to recycle glycolytic NAD+ [19]. Alternatively, altered TAG metabolism in our model may be a consequence of decreased Palmitoyl-CoA utilization for sphingolipid synthesis. Recently, transgenic PHGDH overexpression was found to significantly reduce hepatic TAG accumulation on a high fat diet [38], further supporting a role for PHGDH in liver TAG metabolism. Fatty acid incorporation into TAGs has been shown to protect against lipotoxicity in many settings [5, 21]. Further work is needed to determine whether shPHGDH livers have mitochondrial impairment, and whether alterations in TAG metabolism protect shPHGDH livers.

## Conclusions

Our study found that PHGDH knockdown has a modest effect on circulating serine in the presence of dietary serine/glycine and does not affect the function or proliferation of adult pancreas, liver, and intestine. However, loss of PHGDH expression reduced liver and serum ceramide levels without increasing the levels of deoxysphingolipids. Further, liver triacylglycerol profiles were altered, with an accumulation of longer-chained, polyunsaturated tails upon PHGDH knockdown. Collectively, these results suggest that PHGDH-derived serine supports liver ceramide synthesis and sustains general lipid homeostasis.

## Supplementary information

**Additional file 1: Supplementary Figure 1.Validation of shRNAs for model development.** NIH3T3 cells expressing Renilla or PHGDH-targeting shRNAs (#1-10) were treated with 1 μg/mL doxycycline for 6 days and PHGDH expression determined by western blot. β-actin is used as a loading control.

**Additional file 2: Supplementary Figure 2. shPHGDH mice have poor knockdown in the spleen**. Western blot analysis of PHGDH, GFP and HSP90 protein levels in spleen of shPHGDH and shREN mice. Mice were placed on a 200 ppm doxycycline diet for 8 months. ns, non-specific band.

**Additional file 3: Supplementary Figure 3.PHGDH knockdown does not affect liver function.** shPHGDH (N = 10) and shREN (N = 10) mouse serum was collected and analyzed by IDEXX (Liver Panel). (A) ALP – Alkaline phosphatase. (B) AST – aspartate transaminase. (C) ALT – alanine transaminase. (D) CK – creatine kinase. (E) – Total albumin. (E) Total bilirubin.

**Additional file 4: Supplementary Figure 4.PHGDH knockdown does not affect brain serine and lipids.** (A) Brain serine quantities of 5- to 9-month-old shREN (N = 15) and shPHGDH (N = 14) mice. Quantities were normalized to mg of tissue. (B) Quantity of individual ceramides in the brain of 5- to 9-month-old shREN (N = 14) and shPHGDH (N = 15) mice. Quantities were normalized to mg of tissue. (C) Volcano plot of lipidomics analysis of shPHGDH (N = 15) brain compared to shREN (N = 15). Significant metabolites are in bold. Triacylglycerol species are indicated in red. (D) Individual TAG species in the brain of shPHGDH mice compared to shREN. Levels are normalized to shREN.

**Additional file 5: Supplementary Table 1.** **Analysis parameters for targeted lipidomics.**

**Additional file 6: Supplementary Table 2. Lipidomics data from Figure****5****A.** Lipidomics analysis of shPHGDH (N = 11) serum compared to shREN (N = 12).

**Additional file 7: Supplementary Table 3. Lipidomics data from Figure****5****B.** Lipidomics analysis of shPHGDH (N = 11) liver compared to shREN (N = 11).

**Additional file 8: Supplementary Table 4. Lipidomics data from Supplementary Figure****4****C.** Lipidomics analysis of shPHGDH (N = 15) brain compared to shREN (N = 14).

## Data Availability

All data generated or analyzed during this study are included in this published article and its supplementary information files. Materials are available from the corresponding author on request.

## Abbreviations

CNS: Central nervous system
dihydrodox: Dihydrodeoxy
dox: Deoxy
NAD+: Nicotinamide adenine dinucleotide
NLS: Neu-Laxova syndrome
PHGDH: d-3-phosphoglycerate dehydrogenase
shPHGDH: shRNA-targeting PHGDH
shREN: shRNA-targeting Renilla luciferase
shRNA: Small hairpin RNA
SPT: Serine palmitoyltransferase
