# Supplementary figures and images for: PHGDH supports liver ceramide synthesis and sustains lipid homeostasis
Source: Cancer Metab. 2020 Jun 15;8:6. doi: 10.1186/s40170-020-00212-x (PMC7294658; doi:10.1186/s40170-020-00212-x)

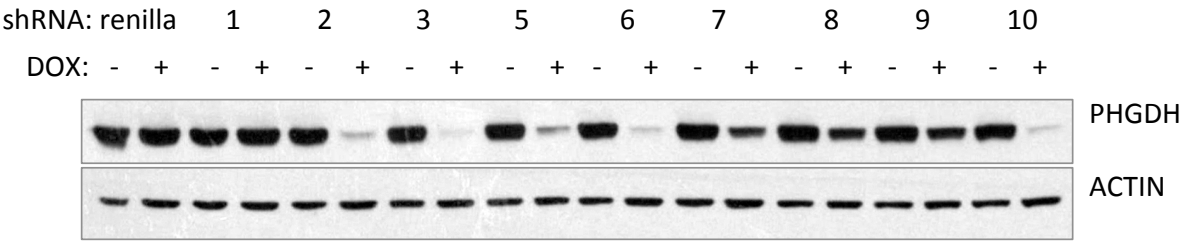

Supplementary Figure 1

Supplement: Supplementary file 1 — Additional file 1: Supplementary Figure 1.Validation of shRNAs for model development. NIH3T3 cells expressing Renilla or PHGDH-targeting shRNAs (#1-10) were treated with 1 μg/mL doxycycline for 6 days and PHGDH expression determined by western blot. β-actin is used as a loading control. [file 40170_2020_212_MOESM1_ESM.pdf]

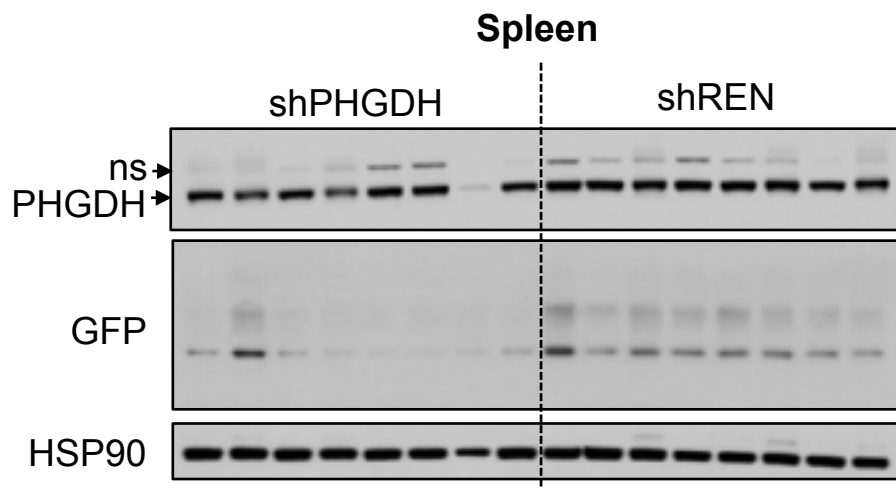

**Supplementary Figure 2**

Supplement: Supplementary file 2 — Additional file 2: Supplementary Figure 2. shPHGDH mice have poor knockdown in the spleen. Western blot analysis of PHGDH, GFP and HSP90 protein levels in spleen of shPHGDH and shREN mice. Mice were placed on a 200 ppm doxycycline diet for 8 months. ns, non-specific band. [file 40170_2020_212_MOESM2_ESM.pdf]

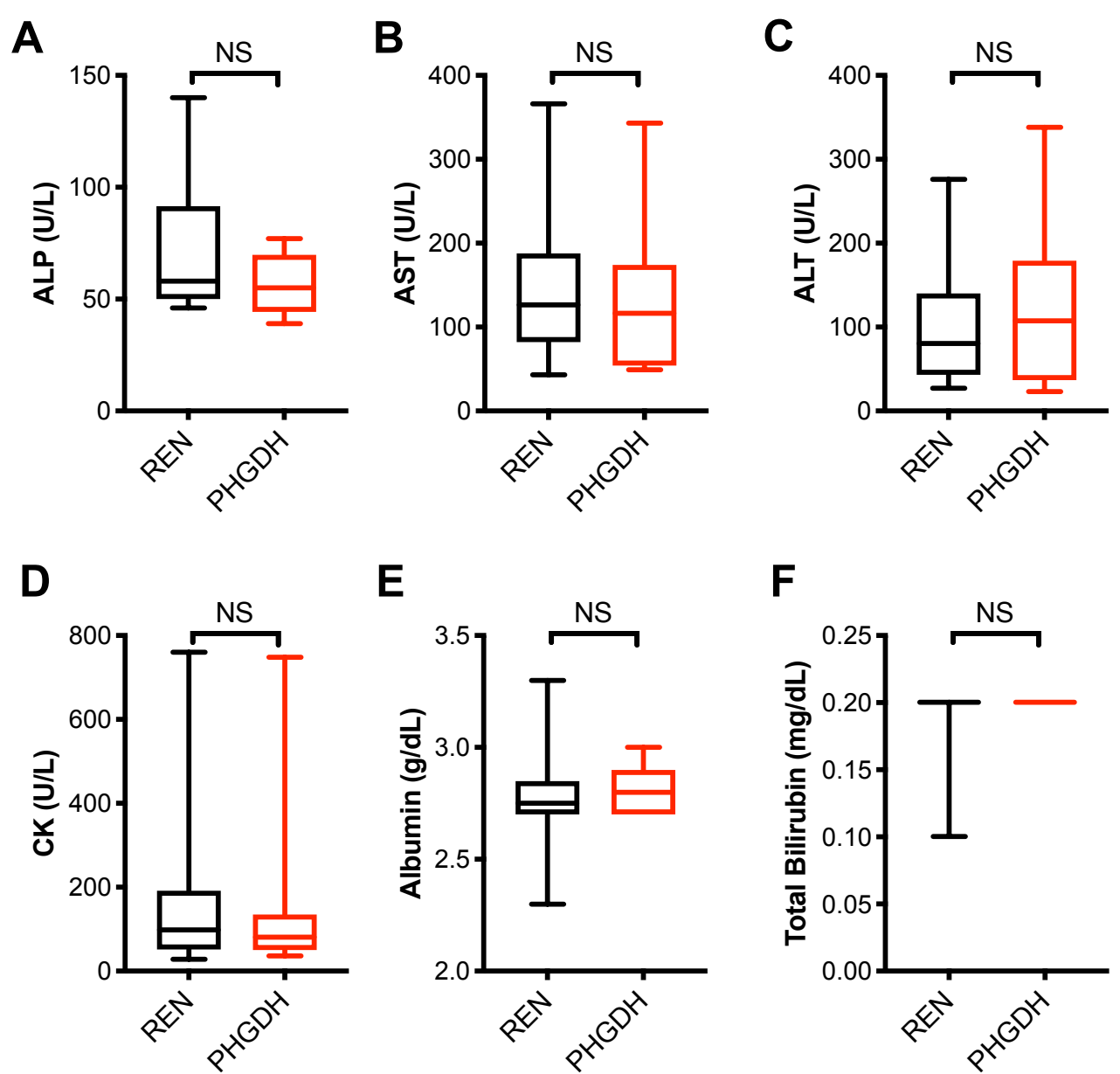

**Supplementary Figure 3**

Supplement: Supplementary file 3 — Additional file 3: Supplementary Figure 3.PHGDH knockdown does not affect liver function. shPHGDH (N = 10) and shREN (N = 10) mouse serum was collected and analyzed by IDEXX (Liver Panel). (A) ALP – Alkaline phosphatase. (B) AST – aspartate transaminase. (C) ALT – alanine transaminase. (D) CK – creatine kinase. (E) – Total albumin. (E) Total bilirubin. [file 40170_2020_212_MOESM3_ESM.pdf]

**A**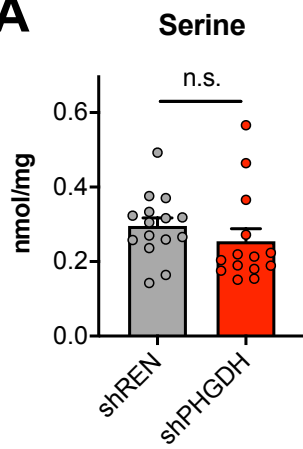**B**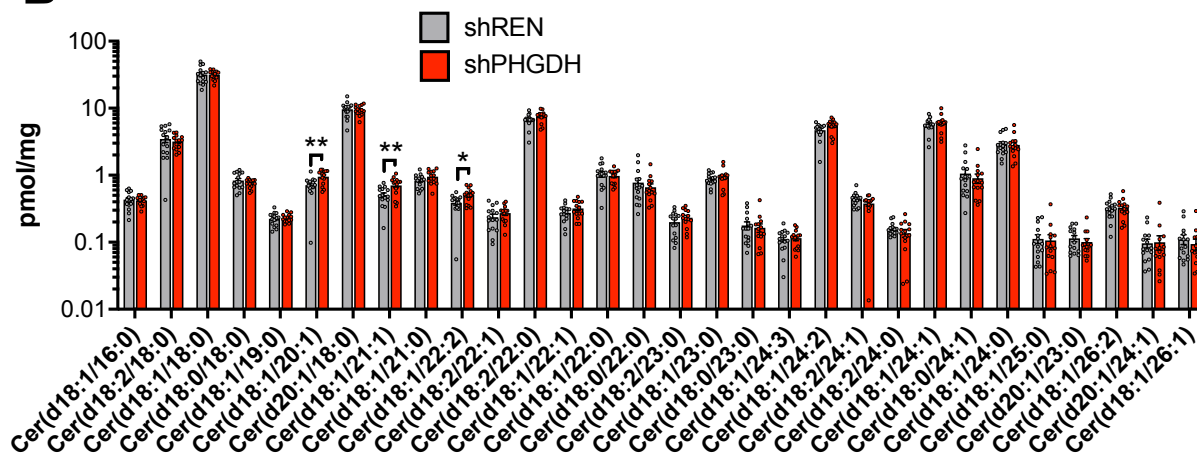**C**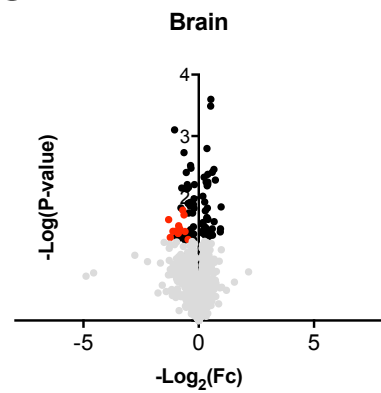**D**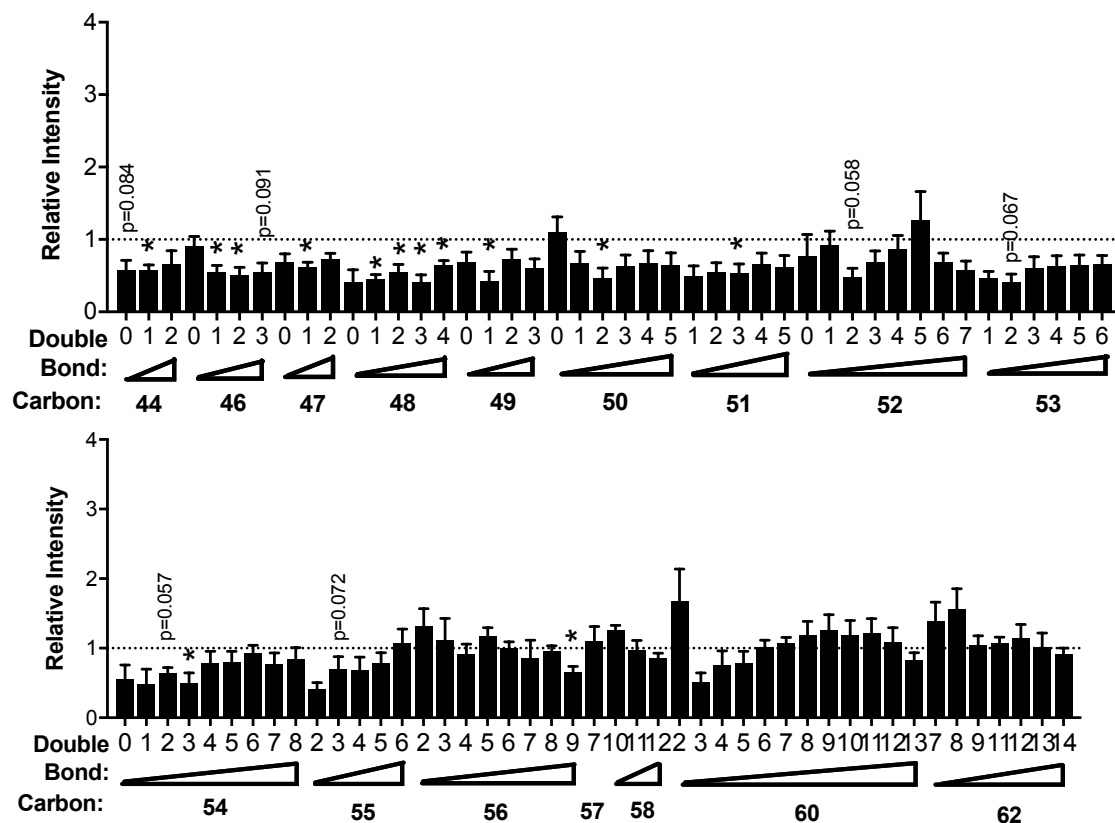

Supplement: Supplementary file 4 — Additional file 4: Supplementary Figure 4.PHGDH knockdown does not affect brain serine and lipids. (A) Brain serine quantities of 5- to 9-month-old shREN (N = 15) and shPHGDH (N = 14) mice. Quantities were normalized to mg of tissue. (B) Quantity of individual ceramides in the brain of 5- to 9-month-old shREN (N = 14) and shPHGDH (N = 15) mice. Quantities were normalized to mg of tissue. (C) Volcano plot of lipidomics analysis of shPHGDH (N = 15) brain compared to shREN (N = 15). Significant metabolites are in bold. Triacylglycerol species are indicated in red. (D) Individual TAG species in the brain of shPHGDH mice compared to shREN. Levels are normalized to shREN. [file 40170_2020_212_MOESM4_ESM.pdf]
